# Construction of a Novel Clinical Stage-Related Gene Signature for Predicting Outcome and Immune Response in Hepatocellular Carcinoma

**DOI:** 10.1155/2022/6535009

**Published:** 2022-07-12

**Authors:** Liu Yang, Long-fei Zeng, Guo-qing Hong, Qing Luo, Xing Lai

**Affiliations:** ^1^Department of Hepatobiliary, Pancreatic and Splenic Surgery, People's Hospital of Luzhou City, Luzhou, China; ^2^Department of Hepatobiliary Surgery, Tongnan District People's Hospital, Chongqing, China

## Abstract

Hepatocellular carcinoma (HCC) with high heterogeneity is one of the most frequent malignant tumors. However, there were no studies to create a clinical stage-related gene signature for HCC patients. Differentially expressed genes (DEGs) associated with clinical stage of HCC were analyzed based on TCGA datasets. Functional enrichment analysis was carried out by the use of stage-related DEGs. Then, the least absolute shrinkage and selection operator (LASSO) regression and univariate Cox regression were performed to reduce the overfit and the number of genes for further analysis. Next, survival and ROC assays were carried out to demonstrate the model using TCGA. Functional analysis and immune microenvironment analysis related to stage-related DEGs were performed. Reverse transcriptase polymerase chain reaction (RT-PCR) and Cell Counting Kit-8 (CCK-8) assays were applied to examine the expression and function of PNCK in HCC. In this research, there were 21 DEGs between HCC specimens with stage (I-II) and HCC specimens with stage (III-IV), including 20 increased genes and 1 decreased genes. A novel seven-gene signature (including PITX2, PNCK, GLIS1, SCNN1G, MMP1, ZNF488, and SHISA9) was created for the prediction of outcomes of HCC patients. The ROC curves confirmed the prognostic value of the new model. Cox assays demonstrated that the seven-gene signature can independently forecast overall survival. The immune analysis revealed that patients with low risk score exhibited more immune activities. Moreover, we confirmed that PNCK expressions were distinctly increased in HCC, and its silence suppressed the proliferation of HCC cells. Overall, our research offered a robust and reliable gene signature which displayed an important value in the prediction of overall survival of HCC patients and might deliver more effective personalized therapies.

## 1. Introduction

Hepatocellular carcinoma (HCC) is one of the most common malignant tumors of the digestive tract [1]. The disease's prevalence and fatality rates are gradually rising, particularly in Asia and Africa [2]. Chronic hepatitis c virus infection, alcohol misuse, obesity, and metabolic syndrome have all been linked to an increased risk of developing HCC [3, 4]. Liver transplantation, percutaneous radiofrequency ablation, and resection are the most common therapies for HCC, with liver resection being the most curative [5, 6]. The 5-year overall survival rate is up to 50%. However, the prognosis for patients in advanced stages is bleak [7, 8]. Therefore, research into biomarkers for cancer prognosis and the development of immunotherapy treatments are essential for enhancing cancer patients' survival.

The stage of HCC samples ranges from stage I to stage IV, with increasing aggressiveness, according to the seventh edition of the American Joint Committee on Cancer (AJCC) cancer staging manual [9]. In the occurrence and development of HCC, as well as the therapy and prognosis of this disease, dynamic alterations in genes are critical [10]. Notably, the prognosis differs greatly depending on when the condition is discovered [11]. With early detection, the 5-year survival rate is 92%, but this declines to 23% by stage IV [12]. Growing evidences have confirmed that clinical stage is an independent prognostic factor for various tumor patients [13]. In addition, the primary tumor, regional lymph nodes, and distant metastasis (TNM) stage have been found to have predictive value for early recurrence [14, 15]. Thus, HCC can be diagnosed and treated more effectively with the identification of clinical stage-related genes.

It is becoming increasingly common to use high-throughput sequencing as a critical tool in the biological sciences, such as for cancer early detection and stage prediction and prognosis prediction [16, 17]. In this study, we utilized the Edger R tool and the TCGA database to find genes that were differentially expressed according to the stage of HCC. Then, we developed a novel model based on critical stage-related genes and explored its association with immune cells. Together, our data revealed a clinical value for the stage-related gene signature and identified a possible marker for HCC prognosis.

## 2. Materials and Methods

### 2.1. Data Collection

TCGA-LIHC and cBioportal for Cancer Genomics provided data on mRNA expression at level 3 and clinical outcomes from 374 LIHC and 50 nontumor samples. It was not necessary to obtain further ethical approval because the data were obtained from a publicly accessible database.

### 2.2. Differences in Gene Expression with Clinical Stage

The data of HCC specimens from the TCGA datasets were divided into two groups: with stage (I-II) and stage (III-IV). Transcripts per million (TPM) was used to standardize the raw count data before a log2 transformation was applied. As a result of this, 19654 protein-coding genes were identified. In order to determine the DEMs, the Limma version 3.36.2 R package was used. An adjusted *P* value of < 0.05 was required for subsequent assays of DEMs with an absolute log2 fold change (FC) of >2.

### 2.3. GO Annotation and KEGG Pathway Enrichment Analyses of DEGs

GO annotation and KEGG pathway enrichment analysis in the Enrichr database revealed the roles of DEGs. The GO terms were comprised of the following 3 divisions: molecular function (MF), cellular component (CC), and biological process (BP).

### 2.4. The Establishment of a Prognostic Model and Prognostic Analysis

LASSO regression methods and univariate assays were applied to find the most closely connected genes with 21 genes strongly associated with clinical stage. There have been seven genes linked to prognosis, and a seven-gene signature has been created. Based on the gene signature's regression coefficients and associated expression values, risk score was computed for every case. Risk scores were calculated using the following formula: Risk score = expressions of Gene 1∗A1 + expressions of Gene 2∗A2 + ⋯+expressions of Gene n∗An. The “survminer” package was used to calculate the cut-off point. We used Kaplan-Meier survival curves and time-dependent receiver operating characteristic curves (ROC curves) to evaluate the model's capacity to differentiate between distinct patient subgroups and its efficiency.

### 2.5. Immunity Analysis and Gene Expression

Our signature was used to compare cell components or cell immune responses between the high-risk and low-risk groups using MCPcounter [18], ESTIMATE [19], CIBERSORT, TIMER [20], and single-sample gene set enrichment analysis (ssGSEA) [21] algorithms. A heat map revealed the variations in immune response under various methods.

### 2.6. Cell Lines and Cell Culture

HCC cells (Huh7, HepG2, HCCLM3, Hep3B, and SMMC-7721) and LO2 cells (as control cells) were bought from Fubo Bio (Haidian, Beijing, China). The cells were cultured in RPMI 1640 medium (Gibco, China) containing 10% fetal bovine serum (FBS, Gibco, Shanghai, China) and 100 units/ml penicillin and streptomycin at 37°C and 5% CO_2_.

### 2.7. RNA Interference

RiboBio (Guangzhou, China) created and made available PNCK's unique siRNA. Using Lipofectamine 3000 transfection reagent (Invitrogen, Shanghai, Pudong, USA), and the manufacturer's instructions were followed for temporary transfection.

### 2.8. Quantitative Real-Time PCR (qPCR)

The total RNA extraction kit was used to extract the total RNA (DP419, Tiangen Biotech, China). Then, using super M-MLV reverse transcriptase, the RNA was converted into cDNA (NG212, Tiangen Biotech). Amplification was performed by 2× Taq PCR MasterMix (KT201, Tiangen Biotech) at the presence of SYBR Green (SY1020, Solarbio, China) as per the users' instructions. For each system, the above operations were performed in triplicate. It was GenScript Co., Ltd. that manufactured the primers, and the primer sequences are provided in Table 1. Amplification of the DNA was carried out using an ExicyclerTM 96 (Bioneer, Korea).

### 2.9. Cell Counting Kit-8 (CCK-8) Assay

The HCC cells were planted in 96-well plates for measuring proliferation ability by CCK-8 test (Dojindo, Kumamoto, Japan). Before adding 10 L of CCK8 (5 mg/mL) to the culture media in each well, cells were grown for 0, 1, 2, 3, or 4 days. A microplate reader measured the absorbance at 450 nm.

### 2.10. Statistical Analysis

Data were analyzed using GraphPad Prism 6.0 Software (GraphPad Inc., San Diego, CA, USA) and R software (Version 3.6.3, The R Foundation for Statistical Computing). When comparing > two groups, the two-way analysis of variance was utilized, whereas the Student's *t*-test was utilized when analyzing differences between just two groups. Plots of Kaplan-Meier curves and log-rank tests were performed to determine whether or not there was a statistically significant difference in OS between the groups. In addition, univariate and multivariate Cox proportional hazard regression analyses were carried out in order to investigate the relationship between risk score and OS. An examination of the sensitivity and specificity of employing the gene signature risk score to predict survival was carried out with the use of the receiver operating characteristic (ROC) analysis. The area under the receiver operating characteristic curve (AUC) was used as an indicator of the accuracy of the prognosis. *P* values were regarded to be statistically significant if they were lower than 0.05 and were two-sided.

## 3. Results

### 3.1. Identification of DEGs in HCC Specimens with Different Clinical Stage

**Figure 1:**
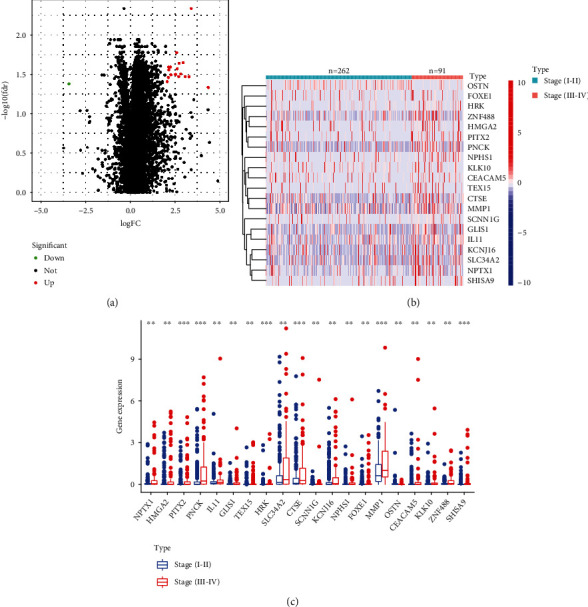
Identification of DEGs in HCC specimens with different clinical stages. (a) Volcano plot of DEGs in HCC specimens with different clinical stage in TCGA datasets. (b) Heat map of DEGs in the TCGA datasets. (c) Expressions of the 21 genes in HCC specimens with different clinical stage.

By the use of TCGA datasets, we firstly examined the genes that exhibited differential expressions in HCC with stage (I-II) and stage (III-IV). The results revealed that there were 21 DEGs between HCC specimens with stage (I-II) and HCC specimens with stage (III-IV), including 20 increased genes and 1 decreased genes (Figure 1(a)). The heat map of differential gene expression reveals the 21 genes that show the most significant differences between the two groups (Figure 1(b)). In addition, the expressing pattern of 21 genes was also shown using histogram (Figure 1(c)).

### 3.2. Functional Enrichment Analysis of DEGs

**Figure 2:**
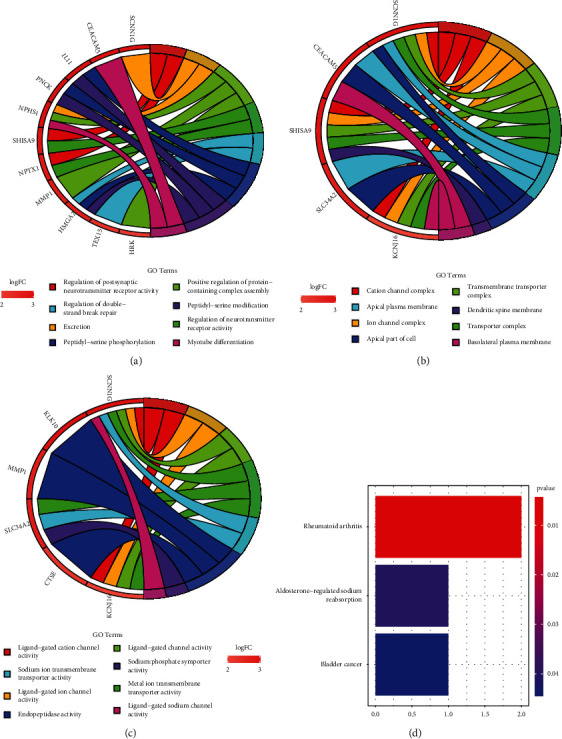
GO and KEGG assays of the 21 genes. Circle plot of (a) BP, (b) CC, and (c) MF assays. (d) KEGG assays.

Enrichment analysis was performed on these 21 genes so that we could have a better understanding of the molecular mechanisms underlying genes that are connected to clinical stages. We observed that the seven genes have important roles in positive regulation of protein-containing complex assembly, peptidyl-serine phosphorylation, peptidyl-serine modification, regulation of postsynaptic neurotransmitter receptor activity, excretion, cation channel complex, ion channel complex, transmembrane transporter complex, transporter complex, apical plasma membrane, metal ion transmembrane transporter activity, endopeptidase activity, ligand-gated cation channel activity, ligand-gated ion channel activity, and ligand-gated channel activity (Figures 2(a)–2(c)). According to the findings of the KEGG study, the 21 genes in question play significant parts in the pathophysiological processes of rheumatoid arthritis, aldosterone-regulated salt reabsorption, and bladder cancer (Figure 2(d)).

### 3.3. Construction of a Prognostic Model in the TCGA Cohort

**Figure 3:**
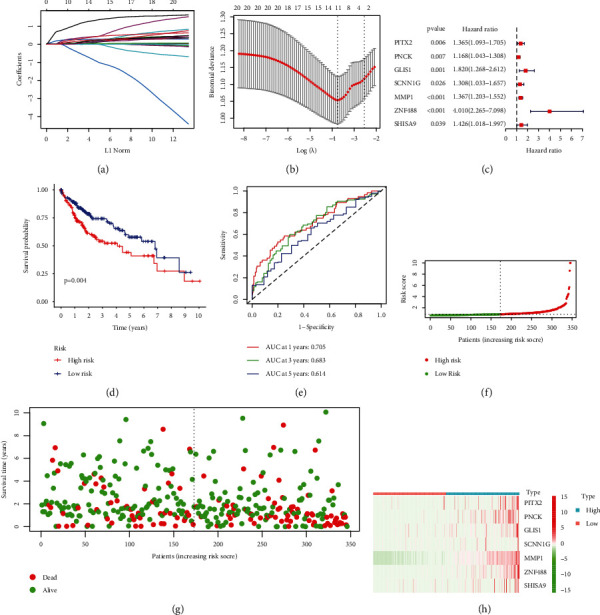
Establishment of a stage-related gene signature in TCGA datasets. (a) Distribution of LASSO coefficients of the 21 stage-related genes. (b) The generated coefficient distribution plots for the logarithmic (lambda) sequence for the selection of the best parameter (lambda). (c) Univariate assays confirmed 7 genes as critical prognostic genes, including PITX2, PNCK, GLIS1, SCNN1G, MMP1, ZNF488, and SHISA9. (d) Kaplan-Meier curves for overall survival. (e) The total survival risk score's ability to forecast future events is supported by time-dependent receiver operating characteristic curves. (f–h) Risk score, survival time, and survival status in TCGA datasets.

**Figure 4:**
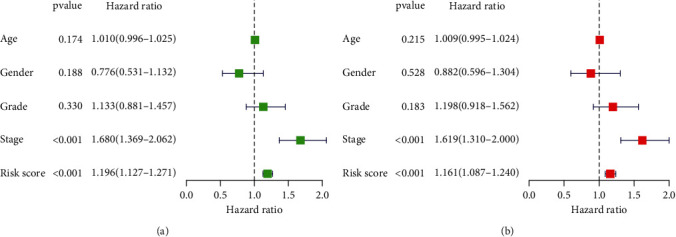
Univariate (a) and multivariate (b) assays were applied to further confirm the prognostic value of the 7-gene signature.

A prognostic model was established by using LASSO and Cox regression analysis on the expression profile of the 21 genes that were discussed earlier in this paragraph. Based on the ideal value of, we were able to identify 13 genes (Figures 3(a) and 3(b)). Further, univariate assays confirmed 7 genes as critical prognostic genes, including PITX2, PNCK, GLIS1, SCNN1G, MMP1, ZNF488, and SHISA9 (Figure 3(c)). Then, we developed a prognostic model, and risk score was computed by the use of the following formula: Risk score = (0.0616)∗PITX2 + (0.0760)∗PNCK + (0.1238)∗GLIS1 + (0.0957)∗SCNN1G + (0.2354)∗MMP1 + (0.7193)∗ZNF488 + (0.1077)∗SHISA9. In accordance with the median value that was used as the dividing line, the patients were classified as belonging to either a high-risk group or a low-risk group. Survival assays indicated that patients with high risk score exhibited a shorter OS (Figure 3(d)). Using time-dependent ROC curves, the predictive ability of the risk score for OS was examined, and AUC achieved 0.705 at 1 year, 0.683 at 2 years, and 0.614 at 3 years (Figure 3(e)). The survival status of all HCC patients based on new signature was shown in (Figures 3(f)–3(h)). We completed univariate and multivariate tests in order to provide additional evidence supporting the diagnostic utility of the 7-gene signature. The results confirmed that risk score and stage are independent predictors of overall survival of HCC patients (Figures 4(a) and 4(b)).

### 3.4. Correlation of Prognosis-Related Genes with TIICs in HCC Patients

**Figure 5:**
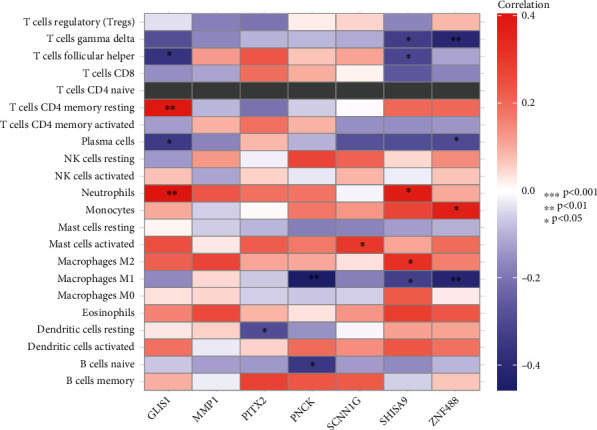
Seven genes, respectively, in relation to the level of immune cell infiltration. ∗*P* < 0.05, ∗∗*P* < 0.01, and ∗∗∗*P* < 0.001.

**Figure 6:**
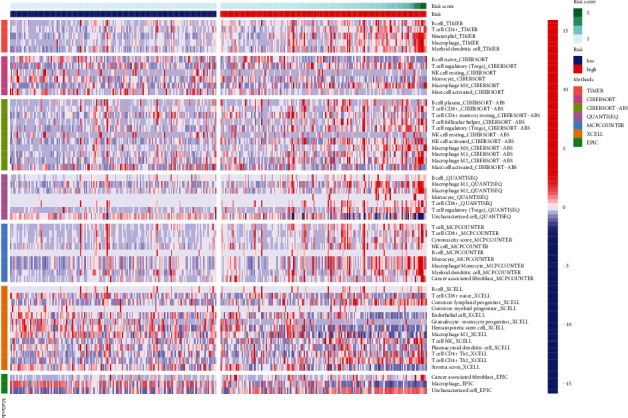
Heat map for immune responses according to five algorithms among low- and high-risk group.

Then, we investigated the link between the seven HCC genes and immune infiltration into the cancerous tissue. Our group observed that PITX2, PNCK, GLIS1, SCNN1G, MMP1, ZNF488, and SHISA9 were significantly correlated with several immune cells, suggesting that the presence of these genes helps to ease the entry of immune cells into HCC, which can help prevent the disease (Figure 5). In addition, Figure 6 displays a heat map of immunological responses that was generated using five algorithms. High-risk score was associated with B cell, T cell CD4+, neutrophil, macrophage, and myeloid dendritic cell.

### 3.5. PNCK Expression Was Upregulated in HCC and Promoted the Proliferation of HCC Cells

**Figure 7:**
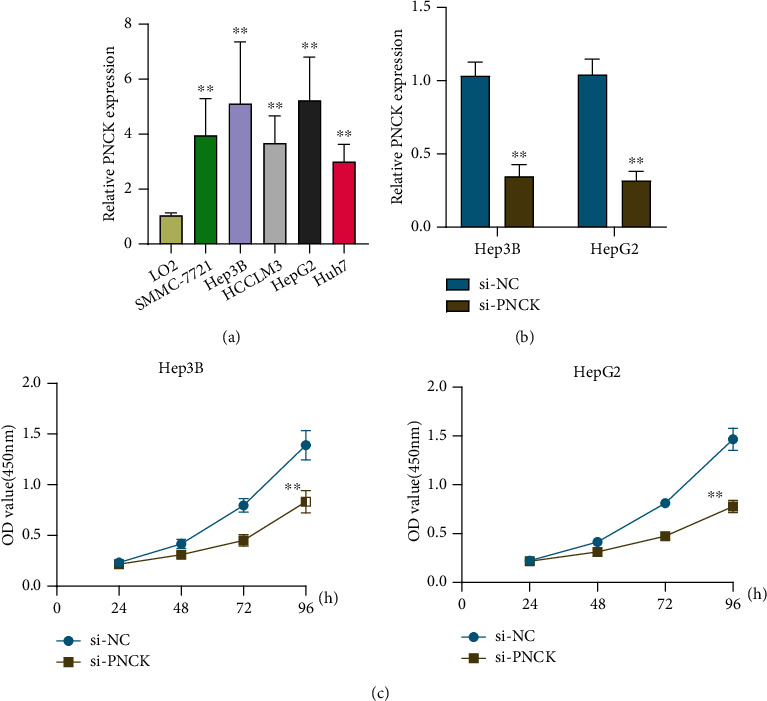
The oncogenic roles of PNCK in HCC progression. (a) RT-PCR was used to examine the expressions of PNCK in four HCC cells and LO2. (b) The expression of PNCK was distinctly decreased in HepG2 and Hep3B cells after the transfection of si-PNCK. (c) Proliferation of HepG2 and Hep3B cells with knockdown of PNCK by CCK-8 assay. ∗∗*P* < 0.01.

Among the seven genes, we focused on PNCK which has been reported to be involved in the progression of several tumors. We performed RT-PCR to examine the expression of PNCK in HCC cells, finding that its expression was distinctly increased in HCC cells compared with LO2 cells (Figure 7(a)). Then, we performed loss-of-function experiments to explore its function. A distinct decrease of PNCK expression was observed in Hep3B and HepG2 cells transfected with si-PNCK, which was demonstrated using RT-PCR (Figure 7(b)). Further, CCK-8 experiments revealed that PNCK knockdown distinctly suppressed the proliferation of Hep3B and HepG2 cells (Figure 7(c)).

## 4. Discussion

The incidence of HCC makes it the fifth most prevalent type of tumor in the world and the third most common reason for death from tumor [22]. Because there are currently no reliable and accurate prognostic biomarkers or models, the clinical prognosis of HCC patients continues to be a primary area of focus for research and development [23, 24]. Over the course of the last ten years, a substantial amount of research has demonstrated that functional genes play an essential part in the progression of tumor growth [25–27]. It has been common knowledge among us that the clinical stage of HCC patients is connected to their overall prognosis. The prognostic model that is based on stage-related genes has been described very infrequently up until this point.

In the course of the last few decades, a great number of studies have investigated various prognostic models for HCC patients [28–30]. In this research, we examined the differences in gene expressions between HCC specimens with stage (I-II) and HCC specimens with stage (I-II) to identify potential gene biomarkers using the TCGA database. In order to construct a risk model that can accurately predict HCC prognosis, the differentially expressed genes were filtered, and univariate, Lasso, and multivariate Cox analyses were performed. We identified seven genes: PITX2, PNCK, GLIS1, SCNN1G, MMP1, ZNF488, and SHISA9. High expression levels of PITX2, PNCK, GLIS1, SCNN1G, MMP1, ZNF488, and SHISA9 were relevant to a poor outcome in HCC cases. Patients with HCC were separated into a high-risk group and a low-risk group by the use of the gene-based risk scoring predictive model. We discovered that patients who were classified as having a high risk of developing the disease had a much worse overall survival rate than their low-risk peers. In addition, it is also worth noting that, in terms of AUCs for the prognostic model's ROC curve, the seven-gene signature showed an excellent performance for the prediction of 1-year, 2-year, and 3-year survival rates. Multivariate assay confirmed that risk score is an independent predictor of overall survival of HCC patients. Our findings suggested the new signature had significant implications in the prediction of clinical outcome of HCC patients.

In the tumor microenvironment (TME), malignant cells live alongside stromal and immune cells [31]. Tumor progression and immunotherapeutic response are dependent on the presence of immune cells within the tumor [32]. New prognostic indicators can be discovered by analyzing the tumor-infiltrating population to learn more about the mechanisms that underlie anticancer immune responses [33, 34]. Methods for determining the presence of tumor-infiltrating immune cells rely heavily on immunohistochemistry (IHC) and flow cytometry [35, 36]. The volume of tumor tissue that must be used and the number of cell types that may be measured at the same time are two of the numerous limitations that prevent these procedures from being completely accurate. Tumor immune state can be assessed using computational methods applied to gene expression profiles of large samples of tumor tissue. In this study, analysis of seven genes in HCC and immune infiltration was carried out. Our assays revealed that PITX2, PNCK, GLIS1, SCNN1G, MMP1, ZNF488, and SHISA9 were significantly correlated with several immune cells, suggesting that these genes may influence the functions of the immune infiltration of HCC. In addition, we also observed that high-risk score was associated with B cell, T cell CD4+, neutrophil, macrophage, and myeloid dendritic cell. HCC's immunological landscape has previously been studied by focusing on the above immunes. Macrophages may have a role in cancer spreading as well as immune suppression, while Tregs may play a role in promoting tumor formation. Both an increased and diminished immune response can have a positive or negative impact on the efficacy of immunotherapy. Overall, it is possible that the discrepancies in survival across patient groups indicated by our signature are due to a dysregulated immunological environment. Clinical integration of 7-gene signature needs to be tested directly, though appears promising from these initial results.

Many studies have reported that some functional genes may be involved in tumor growth of HCC cells. In this study, we focused on PNCK. In recent years, several studies have reported the roles of PNCK dysregulation in several types of tumors. For instance, proliferation, clonal growth, cell-cycle progression, and resistance to trastuzumab were all boosted in SkBr3 cells when PNCK was overexpressed [37]. Chen et al. reported that in human metastatic NPC samples, both PNCK mRNA and protein expression were shown to be increased. The NF-B/VEGF signaling pathway was activated in vitro by upregulation of PNCK, which increased NPC cell motility, invasion, and development of lung metastases [38]. In addition, Cho and his group showed that high levels of PNCK expression in HCC were linked to a worse prognosis [39]. However, its specific function in HCC remained largely unclear. In this study, we found that PNCK expression was distinctly upregulated in HCC cells, which was consistent with previous findings and the above results from TCGA datasets. Moreover, the data of CCK-8 indicated that knockdown of PNCK distinctly suppressed the proliferation of HCC cells. Our findings may explain the reason that patients with high PNCK showed a poor prognosis.

This study also has limitations. First, the TCGA database was used to analyze the data. The sample size was too little, so we could not conduct random grouping to evaluate and verify the results. Second, in order to better understand the role of the stage-related genes in the molecular mechanism, further investigations with in vitro and in vivo were needed.

## 5. Conclusion

PITX2, PNCK, GLIS1, SCNN1G, MMP1, ZNF488, and SHISA9 were all expressed in HCC patients, and the seven-gene signature they produced exhibited a strong ability to predict patient survival. In addition, the study found a link between tumor immune infiltration and risk score. These findings offer a new perspective on the development of new HCC-targeted medicines and the improvement of immunotherapy.

## Figures and Tables

**Table 1:** The primer sequences included in this study.

Genes	Primer sequences (5′-3′)
PNCK: forward	GAAACACACGGAGGACATCAG
PNCK: reverse	GAGCACTGCGATCTCGTTCT
GAPDH: forward	GGAGCGAGATCCCTCCAAAAT
GAPDH: reverse	GGCTGTTGTCATACTTCTCATGG

## Data Availability

The data used to support the findings of this study are available from the corresponding author upon request.
